# MSPAN: A Memristive Spike-Based Computing Engine With Adaptive Neuron for Edge Arrhythmia Detection

**DOI:** 10.3389/fnins.2021.761127

**Published:** 2021-12-15

**Authors:** Jingwen Jiang, Fengshi Tian, Jinhao Liang, Ziyang Shen, Yirui Liu, Jiapei Zheng, Hui Wu, Zhiyuan Zhang, Chaoming Fang, Yifan Zhao, Jiahe Shi, Xiaoyong Xue, Xiaoyang Zeng

**Affiliations:** State Key Laboratory of ASIC and System, School of Microelectronics, Fudan University, Shanghai, China

**Keywords:** spike-based, neuromorphic computing, memristive, computation in memory, arrhythmia detection

## Abstract

In this work, a memristive spike-based computing in memory (CIM) system with adaptive neuron (MSPAN) is proposed to realize energy-efficient remote arrhythmia detection with high accuracy in edge devices by software and hardware co-design. A multi-layer deep integrative spiking neural network (DiSNN) is first designed with an accuracy of 93.6% in 4-class ECG classification tasks. Then a memristor-based CIM architecture and the corresponding mapping method are proposed to deploy the DiSNN. By evaluation, the overall system achieves an accuracy of over 92.25% on the MIT-BIH dataset while the area is 3.438 mm^2^ and the power consumption is 0.178 μJ per heartbeat at a clock frequency of 500 MHz. These results reveal that the proposed MSPAN system is promising for arrhythmia detection in edge devices.

## Introduction

Recently, remote healthcare monitoring has received increasing attention for biomedical applications in edge devices. Driven by the increasing performance of artificial intelligence (AI), especially deep learning (DL), the healthcare monitoring applications has spread to various aspects including early warning, diagnosis, treatment, and prognosis (Sodhro et al., 2018; Alam et al., 2019; Patan et al., 2020; Pustokhina et al., 2020). Because such applications are usually deployed in edge devices where computing and memory resources are extremely limited, energy-efficient DL systems are highly required with corresponding software and hardware implementations.

One of the most valuable edge biomedical applications is remote monitoring of cardiovascular disease, which has become one of the most serious threats of human health nowadays (Wilkins et al., 2017; Zhou et al., 2018; Mehra et al., 2020). Evidence shows that the occurrence of cardiac accidents can be predicted by interpreting the ECG signal in advance, so as to provide valuable time for the intervention of emergency means (Ince et al., 2009; Purushothaman et al., 2014; Vafaie et al., 2014; Rajpurkar et al., 2017; Zhang et al., 2018; Attia et al., 2019; Hannun et al., 2019). Compared with the bedside ECG monitoring devices, the wearable ones are more suitable for remote long-term and real-time monitoring owing to convenient setup, thus attracting extensive research. In the scene of home monitoring, the monitoring device needs to collect the ECG signal constantly without medical staff at side (Clark et al., 2018; Ozkan et al., 2019). Therefore, the main challenge to design such systems is to make the detection of abnormal ECG signals automatic, low-power, and real-time (Yasin et al., 2017; Ai et al., 2018).

The biologically inspired spiking neural network (SNN) has proven to be powerful in computing with low hardware costs, providing a promising solution to the challenges mentioned above. However, the existing SNN training algorithms such as tempotron (Iyer and Chua, 2020), spiking-time dependent plasticity (STDP) (Pu and Cook, 2015), remote supervised method (ReSuMe) (Ponulak and Kasiński, 2010), and SpikeProp (Bohte et al., 2000) suffered from remarkable computation cost and performance loss compared with deep neural networks (DNNs). To achieve a balance between the computation cost and the training complexity, a spiking convolutional neural network (Spiking-CNN) was proposed (Tian et al., 2021) to deal with EEG signals. A traditional CNN was first trained and then transformed to SNN with the trained weights. However, Spiking-CNN separated the training and the inferring procedures of SNN, and this may bring an uncertain degradation of the network performance. To design a spike-based training method, Wu et al. (2018) proposed the back propagation for spiking networks in both spatial and temporal domain, but this method failed to improve both the performance and the energy efficiency. Therefore, novel techniques using intrinsically spiking-based algorithms are still pursued to develop a hardware-friendly and high-performance SNN.

Recently, the progress of resistive random-access memory (RRAM), which is a two-terminal device applying memristors to realize resistive switching, provides potentials for energy-efficient neural network deployment (Guo et al., 2019). Due to the energy-efficient features of memristors, attempts utilizing memristors to build synapses (Wang et al., 2017) and neurons (Wang et al., 2018) have been made, and achieved great progress. The conductance of RRAM can be modulated by electrical pulses either through a variably conductive filament or through the migration of oxygen vacancies (Milo, 2020; Xiao et al., 2020). In addition, RRAM has attractive features, such as high scalability, low consumption power, fast write/read speed, stable storage, and multi-value tune ability. Moreover, RRAM can be applied to in-memory computing for neural network deployment and provides appreciable potential to break the memory wall encountered by conventional von-Neumann architecture.

Multiplication and accumulation (MAC) operation, which is the major computation type in neural network processing, usually dominates the energy consumption and the latency in a hardware system. Computing in memory (CIM) embeds the MAC calculation in memory array (Verma et al., 2019) with a mixed-signal computing paradigm, which is promising to address the computational energy and performance bottlenecks encountered by conventional von-Neumann architecture. Besides, the MAC operations can be performed with high parallelism. The input vector activates multiple rows at a time and the dot product is the sum of column currents weighted by the conductance of memory cells (Strukov et al., 2009; Hu et al., 2017). However, large analog-to-digital converters (ADCs) are always required at the side of the array, which may bring huge overheads in area and energy.

Memristor has shown excellent performance in simulating both spike-based neurons and synapses in hardware, and the use of SNN is expected to further reduce the computational power consumption. However, there have been few studies on RRAM-based SNN. Compared with RRAM-based artificial neural network (ANN), the RRAM-based SNN proposed by Tang et al. (2015) requires only 1/7 power consumption with a slight accuracy degradation (∼2%). However, it fails to consider the non-ideal circuit conditions such as interconnection effects and non-linear effects, and the coding mechanism of SNN also has not been properly explored. For the first time (Valentian et al., 2019), integrated the spike neural network by combining analog neurons and RRAM-based synapses to implement a perceptron design. However, the simple hardware structure can only accommodate a single fully connected layer, which has a poor classification accuracy of 84% on MNIST. Zhang et al. (2020) implemented a temporal coding SNN to recognize the Olivetti face patterns and achieved a better energy efficiency. However, the hardware scale is too small to store enough weights, making it difficult to accomplish complex tasks. To sum up, the works mentioned above are all based on the traditional image recognition database and may not perform well for healthcare applications like arrhythmia detection.

In this work, to overcome the above-mentioned problems, a memristive spike-based computing engine with adaptive neuron – MSPAN is proposed by software and hardware co-design to realize an energy-efficient approach for biomedical application in edge devices. Our contributions lie in the following aspects:

(1) An energy-efficient deep integrative spiking neural network (DiSNN) is proposed as well as the training and inferring strategies. The computation complexity can be largely reduced compared to CNN-based methods while keeping high performance.
(2) A memristor based ADC-free CIM architecture is proposed for inference with threshold adaptive leaky integrate and fire (LIF) neuron module to mimic the function of human brains.
(3) The 4-bit signed mapping method and the corresponding weighted current mirror addition (WCMA) circuit are proposed for the proposed DiSNN to further reduce the area and power consumption overheads.
(4) The proposed memristive system is applied to dealing with biomedical signals in edge devices and achieves both high accuracy and energy efficiency in ECG-based arrhythmia detection tasks.

The remaining sections of this paper are organized as following. Section “Materials and Methods” introduces the key theories and modules of the proposed system. Experimental results and discussions are described in section “Results and Discussion.” Section “Conclusion” concludes this paper.

## Materials and Methods

In this section, the key modules and the corresponding theories of the proposed MSPAN are described in detail, including the spike encoder design, adaptive LIF neuron model, the structure of DiSNN as well as its computing strategy, the memristor based CIM architecture design, the memristor based neural dendrites, the signed weight mapping scheme with corresponding circuits and the threshold-adaptive neuron circuit design.

### Poisson Spike Encoder Design

A spike encoder transforms the input data to the spike sequences that can be processed by SNNs. In this study, a Poisson spike encoder is designed based on the Poisson random number generation. The encoding process can be presented as


(1) $$\begin{array}{c} \delta_{i}=\left\{ \begin{array}{c} 1,X_{i}<I_{i}\\ 0,X_{i}=I_{i} \end{array}\right. \end{array}$$

where the subscription *i* stands for the i-th element in tensor I and X. I is the input tensor, and X is a randomly generated tensor, whose values of elements distributes uniformly in the range of (0, 1). For each time step *t* in the time window *T*, the encoder would generate a spike tensor δ_*t*_. According to the definition of Poisson distribution, the total number of spikes at one position in the whole time window would follow Poisson distribution approximately when time window *T* is large enough. That is the reason why this encoder was named Poisson encoder.

The mean value of spikes generated at position *i* can be obtained through


(2) $$\begin{array}{c} \lambda =TI_{i} \end{array}$$

### Adaptive Leaky Integrate and Fire Neuron Model

**FIGURE 1:**
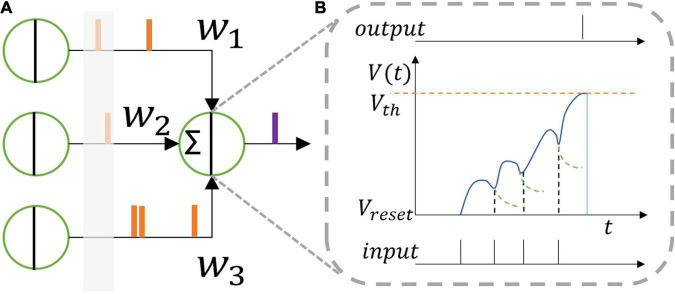
**(A)** Leaky integrate and fire neuron model and **(B)** its function. One neuron receives spikes from the former layer which are integrated inside the neuron membrane. The membrane voltage leaks when no spike comes. When the membrane voltage reaches the threshold voltage, the neuron outputs a spike to the next layer.

Leaky Integrate and Fire model is often used to emulate neuronal behaviors (Lapique, 1907). Inspired by natural behavior patterns of biological neurons, the proposed adaptive LIF neuron model in this work can avoid from being constantly active or inactive. Otherwise, the network performance may be hugely degraded. Besides, a self-modulated function is employed in this work to ensure that the pre-synaptic stimulus of each neuron be constantly non-negative, thus making the model more biological plausible and perform better. Mathematically, the self-modulated function can be expressed by Eqs 3–7 as below:


(3) $$\begin{array}{c} \tau \frac{du}{dt}=-u+IR_{0},u<V_{th} \end{array}$$

(4) $$\begin{array}{c} u=0,u\geq V_{th} \end{array}$$

(5) $$\begin{array}{c} o=\left\{ \begin{array}{c} 1,u\geq V_{th}\\ 0,u<V_{th} \end{array}\right. \end{array}$$

(6) $$\begin{array}{c} I_{0_{LIF}}=\sum_{j=1}^{l}w_{j}o_{j}^{prev} \end{array}$$

(7) $$\begin{array}{c} I=\left\{ \begin{array}{cc} I_{0_{LIF}}, & I_{0_{LIF}}\geq 0\\ 0, & I_{0_{LIF}}<0 \end{array}\right. \end{array}$$

where *u* denotes the membrane potential of the neuron, *R*_0_ the unit resistance (equals 1 in value), τ the time constant, *I*0_*LIF*_ the weighted sum of all the spike inputs which stand for original pre-synaptic stimuli from the previous layer at the current time step, *l* the length of the last layer, *I* the modified pre-synaptic stimulus, and *o*, *o^prev^* the output spike of the current layer and the previous layer, respectively. *V_th_* is the threshold voltage that is adaptive for neurons in every layer of the proposed SNN structure, as discussed in the next section. Whenever the membrane potential of a neuron exceeds the threshold voltage, it will generate a spike, and then reset its membrane potential to the reset voltage, as shown in Figure 1.

The equations above give the differential form of the function of a LIF neuron model. To train the network iteratively, the model needs to be transformed into an iterative form. From Eq. 3, using Euler method, we can obtain that


(8) $$\begin{array}{c} u_{t+1}=\left(1-\frac{dt}{\tau }\right)u_{t}+IR_{0} \end{array}$$

where *u*_*t*1_ − *u*_*t*_ = *du*, and the subscript denotes the time step *t*. Define


(9) $$\begin{array}{c} k_{\tau }=1-\frac{dt}{\tau } \end{array}$$

as the decay constant, and the iterative form can be expressed as below:


(10) $$\begin{array}{c} u_{t+1}=k_{\tau }u_{t}+IR_{0}. \end{array}$$

### Deep Integrative Spiking Neural Network

**FIGURE 2:**
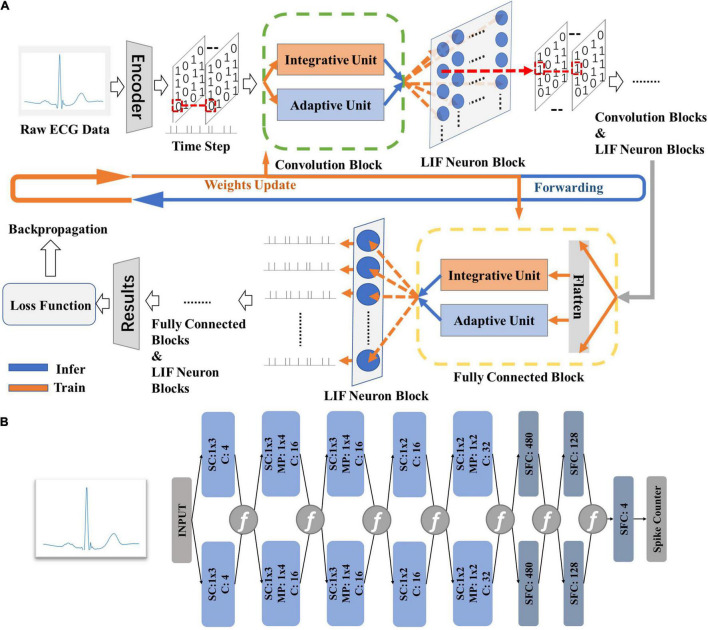
The theory of the proposed DiSNN. **(A)** The computing strategies of DiSNN; **(B)** the model structure of proposed in this work, where “SConv” is spike-based convolution layer, “MP” is max-pooling layer, and “C” stands for channel.

Figure 2B gives the model structure of the proposed DiSNN topology. The DiSNN topology consists of an input layer, five spike-based convolution (SConv) layers, two spike-based fully connected (FC) layers and an output layer. Two max-pooling layers are inserted between the SConv layer and the following one in the second and the fifth layers, respectively. To provide convenience for hardware design, single-dimension (1-D) kernels are employed to extract features from the raw ECG data.

**FIGURE 3:**
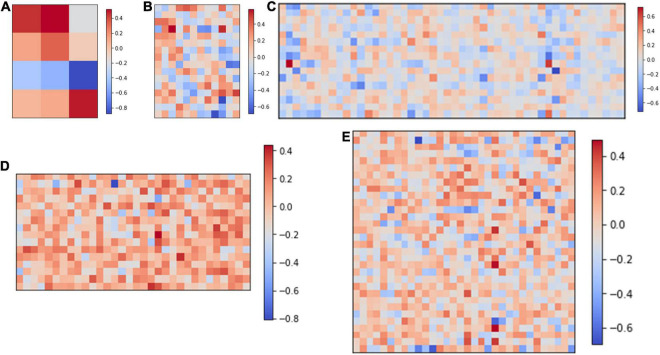
Weights of the five spike-based convolution blocks. **(A)** SConv1, 4*1*1*3; **(B)** SConv2, 16*4*1*3; **(C)** SConv3, 16*16*1*3; **(D)** SConv4, 16*16*1*2; and **(E)** SConv5, 32*16*1*2.

Figure 2A shows the computing strategy of DiSNN. The features of the input ECG sample are interpreted as spike sequences at each time step by the spike encoder. Each SConv or FC block of DiSNN has an integrate unit (IU) and an adaptive unit (AU). The IU integrates the information from the former block while the AU adapts the information to extract the true features against the noise. The update of membrane potential in the LIF neuron is realized jointly by IU and AU at every time step. Figure 3 shows the weight distribution of the five SConv blocks of DiSNN, which is expressed in the format of SConv − number of the block, output channel × input channel × kernel height × kernel width.

At every time step, the output neurons of DiSNN may be activated as needed. A spike counter records the activation number of all the output neurons over all time steps and the one with the highest average activating frequency tells the results of classification. All the data transferred between DiSNN blocks is in single-bit (0 or 1) form, which only requires accumulation operations to accomplish the computing process. The elimination of multiplication operations can significantly reduce computation complexity and potential hardware costs. Therefore, the proposed DiSNN is more hardware-friendly, promising for low-cost edge applications.

### Training and Inferring Method

In the proposed DiSNN, the data flows simultaneously in both temporal and spatial forms. A back propagation algorithm has been given to train spiking neural networks in both domains (Wu et al., 2018). To take both time domain (TD) and spatial domain (SD) into account, the training framework is provided in the following steps.

Assuming function L as the loss function, the gradient descent of each layer is determined as follows. L is presented in Eq. 11:


(11) $$\begin{array}{c} L=\frac{1}{2M}\sum_{m=1}^{M}{\left|y_{m}-\frac{1}{T}\sum_{t=1}^{T}o_{m,t}\right|}^{2} \end{array}$$

where the subscript *m* denotes the *m*th sample, and *t* the *t*th time step. *M* is the number of samples in one batch, and *o*, *y* the output spikes and corresponding labels, respectively. By utilizing the chain rule in *n*th layer and *t*th time step, the gradients can be obtained through Eqs 12, 13


(12) $$\begin{array}{c} \frac{\partial L}{\partial u_{i,t,n}}=\frac{\partial L}{\partial o_{i,t,n}}\frac{\partial o_{i,t,n}}{\partial u_{i,t,n}}+\frac{\partial L}{\partial o_{i,t+1,n}}\frac{\partial o_{i,t+1,n}}{\partial u_{i,t,n}} \end{array}$$

(13) $$\frac{\partial L}{\partial o_{i,t,n}}=\sum_{j=1}^{l_{n+1}}\frac{\partial L}{\partial o_{i,t,n+1}}\frac{\partial o_{j,t,n+1}}{\partial o_{i,t,n}}\frac{\partial L}{\partial o_{i,t+1,n}}\frac{\partial o_{i,t+1,n}}{\partial o_{i,t,n}}$$

where *l*_*n*+1_ denotes the total number of neurons in the *n* + 1th layer. Note that the mathematical form of a spike is a singularity function, i.e., the Dirac function, and cannot be differentiated. To make the partial difference of an output spike computable on hardware, a rectangular pulse function in Eq. 14


(14) $$\begin{array}{c} h\left(u\right)=\left\{ \begin{array}{c} a,\left|u-V_{th}\right|\leq \frac{a}{2}\\ 0,\left|u-V_{th}\right|>\frac{a}{2} \end{array}\right. \end{array}$$

is used to replace the Dirac function, and the length of pulse a stands for a hyper-parameter. Applying the functions of LIF neuron models described in section “Adaptive Leaky Integrate and Fire Neuron Model” to Eqs 7, 8, the gradient of weights can be expressed in Eq. 15


(15) $$\begin{array}{c} \frac{\partial L}{\partial w_{n}}=\sum_{t=1}^{T}\frac{\partial L}{\partial u_{t,n}}o_{t,n-1} \end{array}$$

It is worth noting the fact that a decline in accuracy might occur because of the “Spiking Stall,” which means some neurons keep constantly active through all the time steps and lose the ability to send information to the next layer. In DiSNN, a hardware-friendly method is proposed to solve this problem, i.e., the AUs.

Parallel neuron layers named auxiliary inhibitory layers are assumed for the main structure of the network as shown in Figure 2. Each of the auxiliary inhibitory layers has the same feature with the corresponding active layer and can also be trained. When updating the membrane potential, the weighted sum of all the spike inputs of the original pre-synaptic stimulus I_0*LIF*_ is shown in Eq. 16


(16) $$\begin{array}{c} I_{0_{LIF}}=\sum_{j=1}^{l}\left(w_{j}o_{j}^{prev}-\beta w_{j}^{a}o_{j}^{prev}\right) \end{array}$$

where β is a hyper parameter controlling the intensity of the negative feedback. By constructing the auxiliary inhibitory layers, even if the previous layer sends out an intense stimulus, the rise of the membrane potential will remain within a reasonable range, solving the problem of “Spiking Stall.”

As for the inferring process, the membrane potentials of adaptive LIF neurons of each layer is calculated at every time step and the neuron fires a spike when reaching the threshold. The output neuron accumulates the output spikes over all the time steps. The classification result is given by the index of the output neuron with the highest activation frequency on average.

### Computation in Memory Architecture

**FIGURE 4:**
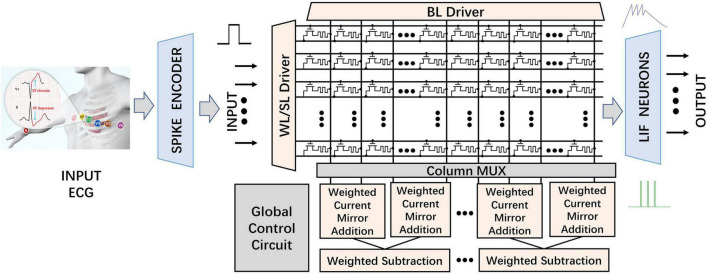
Structure of the proposed MSPAN system (“WL” stands for word line; “SL” stands for source line; “BL” stands for bit line; and “MUX” stands for multiplexer).

The overall hardware architecture of the proposed MSPAN system for inference consists of the CIM structure and the neuromorphic circuits, as shown in Figure 4. Compared with conventional architectures, CIM structure is well known to perform the MAC operations with high energy efficiency and low area consumption (Chi et al., 2016; Shafiee et al., 2016).

The inference phase of the trained DiSNN network is deployed to the above-mentioned architecture, where the 32-bit floating-point (FP32) synaptic weights are quantized, mapped, and loaded into 4 neighboring RRAM cell in a single row in advance. The RRAM cells acting as biological synapses receive the spike-based input data streams from the Spike Encoder based on Poisson distribution as presynaptic input spikes.

In the RRAM-based synaptic array, the word line (WL) is connected to the gate of corresponding transistor to control the on and off of the transistor in a one-transistor-one-RRAM (1T1R) cell as shown in Figure 4. The source line (SL) is connected to the source of the transistor. The upper electrode of RRAM is attached to the bit line (BL), while the lower electrode is serially attached to the drain of the transistor. According to the Kirchhoff’s Law, the output of synaptic array on each BL represents the product of the input and the corresponding weight, in the form of current. During operations, six rows are selected by the WL/SL driver. The input 1-bit pulses are first applied on the SLs and then weighed by the corresponding 4 parallel 1T1R cells. Each BL naturally adds the result of 6 dot products in the form of current according to Kirchhoff’s current law (KCL). The 1-bit inputs and 1-D SConv kernels make it more efficient for network mapping and SConv operations in the proposed CIM-based system. *I_BL_* represents the sum of the result of 6 dot products. Two adjacent groups of BLs (a group contains 4 columns, as defined in subsequent sections) are selected by the column MUX module at one time, and then subtracted in a weighted manner, mimicking the neuromorphic impact of inhibitory neurons on active neurons. Then, the obtained pre-synaptic stimulus currents are applied to the corresponding threshold-adaptive LIF neurons, whose membrane will sample and integrate the input pulses non-linearly and then fire a spike once the accumulated voltage surpasses V_*threshold*_. A set of latches would record the output of each output neurons. Finally, A digital counter adds up the number of pulses emitted by the output LIF neurons through all the time steps and then the classification result is determined by the neuron with the highest activating frequency on average.

### Memristor Based Neural Dendrites

**FIGURE 5:**
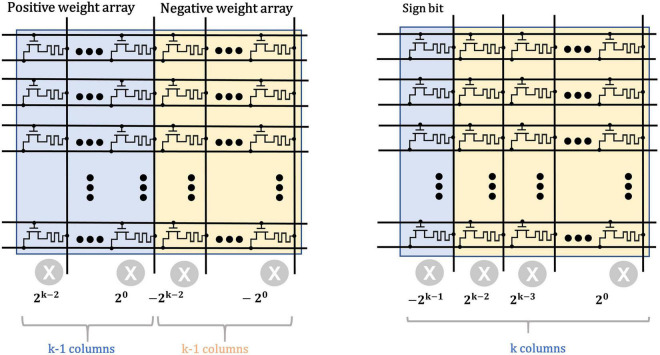
Typical weight storage structure (left) for sign weights compared with the proposed structure (right).

The typical scheme for weight storage (Chi et al., 2016) use two memory arrays to separately store the positive and negative weights of neural networks, as shown in Figure 5. The matrix multiplication operations are first performed separately in each array and then the intermediate results are subtracted in peripheral circuits for the final results. However, this scheme leads to considerable cost in chip area and power consumption. For example, for a signed weight of *k* bits, a total of 2 × (*k* − 1) memristors are required to represent the weight. When it comes to high-precision weights, the overhead of the memristor on the storage array will become tremendous.

In this work, we employ a sign bit to mark the polarity of weights to reduce the area and energy consumption. Besides, the weights are quantized from the FP32 precision to the 4-bit signed fixed-point one which ranges from −8 to +8, to further reduce the size of neural network and the overhead of weight storage. Here, the base {−2^3^, 2^2^, 2^1^, 2^0^} is used to uniformly represent the 4-bit signed numbers and the mapping scheme is shown in Table 1. For example, the decimal (−5)_10_ equals to 1 × (−2^3^) + 0 × 2^2^ + 1 × 2^1^ + 1 × 2^0^, thus it is converted to (1011)_2_.

**TABLE 1:** Signed 4-bit RRAM weight mapping table.

W[3]	W[2]	W[1]	W[0]	Binary value	Value
LRS(1)	HRS(0)	HRS(0)	HRS(0)	1000	−8
LRS(1)	HRS(0)	HRS(0)	LRS(1)	1001	−7
	…				
LRS(1)	LRS(1)	LRS(1)	LRS(1)	1111	−1
HRS(0)	HRS(0)	HRS(0)	HRS(0)	0000	0
	…				
HRS(0)	LRS(1)	LRS(1)	HRS(0)	0110	6
HRS(0)	LRS(1)	LRS(1)	LRS(1)	0111	7

**FIGURE 6:**
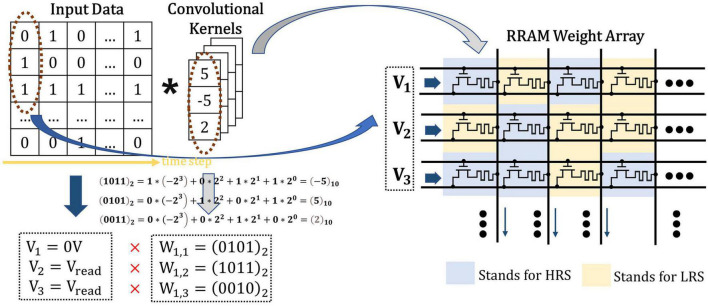
The illustration of spike-based convolution operation with memristor crossbar, where the input data in timestep has been transformed into input voltage and the weights in SConv kernels has been transformed into RRAM resistance states.

The classical 1T1R cells are adopted in the proposed CIM structure to simulate the biological dendrites and store the weights in the form of the conductance of RRAM. Single level cell (SLC) RRAMs are used to implement the network weights, where the high-resistance state (HRS) of SLC RRAM is employed to represent “0,” and the low-resistance state (LRS) for “1,” as shown in Figure 6. Since the floating-point weights have been quantized to a 4-bit signed one, four 1T1R cells are placed in parallel to store one weight in advance during write operation. The 1-bit input data is converted into different voltage levels, “0” for 0V while “1” for *V_read_*.

**FIGURE 7:**
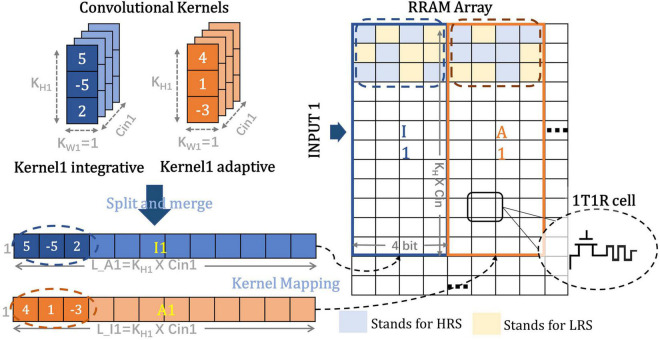
Weight matrixes mapping method for spike-based convolutional layers where K, H, W, L, C, A, and I stand for kernel, height, width, length, channel, adaptive, and integrative, respectively.

As shown in Figure 7, for SConv operation, the 1-D kernels are first split in the input channel direction, then connected and jointed into an array of slender bars, and finally stored in four corresponding parallel columns of the RRAM array. The width of each SConv kernel is set to be 1 to better simulate the biological mechanism and reduce the computational overhead. Therefore, the size of each mapped kernel in the RRAM array is equal to 4 × (*KH*1×*C*_*IN*_1) which can be considered as a group. The odd-numbered groups store the weights of integrative SConv kernels (named as *I_i_*, *i* = 1, 2, 3, …*K*) (suppose *K* as the number of output channels of the layer), while the adjacent even-numbered groups store the corresponding adaptive SConv kernel weights (named as *A_i_*, *i* = 1, 2, 3, …*K*). Group *I_i_* and Group *A_i_* are placed closely together to facilitate the input voltage sharing scheme and the subsequent weighted subtraction operations. For multiple output channels in one SConv bank, different groups in the RRAM array store the weights of different SConv kernels in parallel, which are arranged as *I*_1_, *A*_1_, *I*_2_, *A*_2_,…, *I*_*K*_,*A*_*K*_ in order. As for FC layers with no SConv kernels, the weight matrixes can be directly mapped to the synaptic array based on the proposed 4-bit signed weight mapping scheme.

### Bit-Wise Weighted Current Mirror Addition Circuit

According to the proposed weight mapping scheme, the sign bit is 1 for the negative quantized weights and the corresponding RRAM is in LRS. The current *I_LRS_* of the sign bit needs to be multiplied by the basic weight −2^3^ during bit-merging operation. If the sign bit is 0, which indicates that the weight is positive, the current *I_HRS_* of the sign bit does not need to be included. The synaptic array structure implemented by the 1T1R cells makes the BL currents flow in single direction, thus it requires special processing of the BL current corresponding to the sign bit.

According to the SConv kernel size of DiSNN, the kernel height is two or three. So, we take their least common multiple, six, as the degree of parallelism. Considering that *I_LRS_* ≫ *I_HRS_* [in the simulated RRAM model (Jiang et al., 2016), the on/off ratio >100] and the RRAM synaptic array only processes six rows of MAC operations in parallel at a time, if there exists *I_HRS_* in the BL current for the sign bit, *I_HRS_* can be ignored compared to *I_LRS_*. Therefore, there is no need to design different weighted addition circuits for positive and negative weights, thus greatly reducing the circuit costs.

**FIGURE 8:**
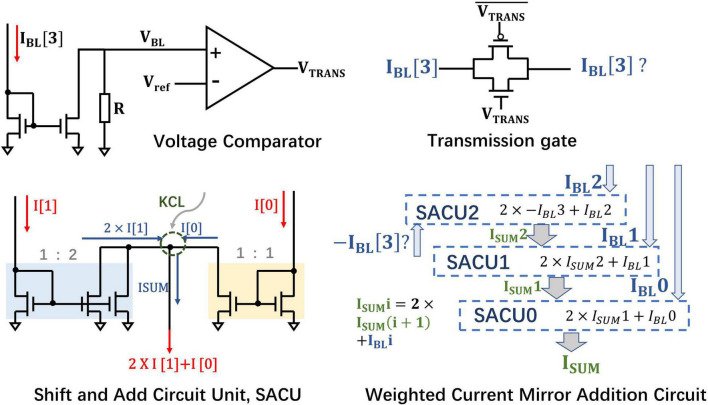
The circuit of bit-wise weighted current mirror addition circuit.

If all of the weights processed in parallel are positive, the sign bit *I*_*BL*_[3] of six sign bits “0” is equal to *6I*_*HRS*_. Although the current is relatively small, when it is directly passed to the weighted current mirror circuit, it will be multiplied by the base of −2^3^ and cannot be ignored, possibly impairing the accuracy of the CIM architecture to a certain extent. Here, a transmission gate is employed to judge whether the *I*_*BL*_[3] of sign bit will be connected to the WCMA circuit, as shown in Figure 8. A voltage comparator compares the voltage between *V_ref_* and *V_BL_* formed by the sampled *I_BL_* through a fixed value resistor, and then produces voltage *V_TRANS_* connected to the gate of the NMOS in the transmission gate. If the six weights processed in parallel are all positive, the BL current of sign bit is supposed to be *6I*_*HRS*_, which is far from turning on the transmission gate, so *6I*_*HRS*_ will not be brought into subsequent calculations. However, once there exists a negative number in the six weights, then *V_BL_* would be larger than *V_ref_* and *V_TRANS_* will be of high voltage, and the transmission gate is expected to open.

Furthermore, since the MAC operation satisfies the Multiplicative Distribution Law and the Associative Law, the value of each 1T1R cell can be naturally added on the BL according to KCL, and then the selected BLs will be weighted and summed. The WCMA circuit is mainly composed of 3 basic Shift and Add circuit mirror circuits (SACU). After 3 iterations, the WCMA circuit is supposed to realize the following Eq. 17:


(17) $$\{\begin{array}{c} I_{sum_{A_{i}}}=\left(-2^{3}\right)\times I_{BL_{A_{i}}}\left[3\right]+2^{2}\times I_{BL_{A_{i}}}\left[2\right]+2^{1}\times I_{BL_{A_{i}}}\left[1\right]\\ +2^{0}\times I_{BL_{A_{i}}}\left[0\right]\;\left(I_{BL_{A_{i}}}\left[3\right]>6I_{HRS}\right)\\ I_{sum_{A_{i}}}=2^{2}\times I_{BL_{A_{i}}}\left[2\right]+2^{1}\times I_{BL_{A_{i}}}\left[1\right]+2^{0}\times I_{BL_{A_{i}}}\left[0\right]\\ \left(I_{BL_{A_{i}}}\left[3\right]=6I_{HRS}\right) \end{array}$$

Equation 17 realizes the dot product of weights and input bit, in the form of analog current in Group *A_i_* (*i* = 0, 1, 2, …) (similar for integrative groups), and illustrates how the current mirror weighted addition circuit merges the currents on the four BLs. Finally, the output currents of auxiliary and inhibitory layers in the same bank will undergo a weighted subtraction operation to realize Eq. 16, mimicking the biological mechanism of lateral inhibition.

### Threshold-Adaptive Leaky Integrate and Fire Neuron Circuit

**FIGURE 9:**
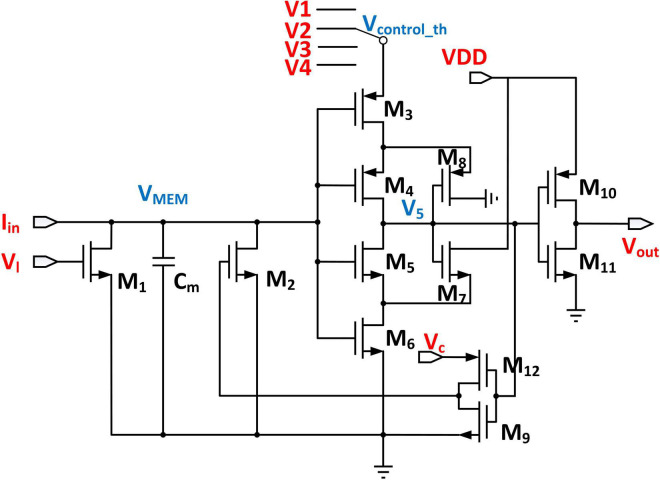
Circuit structure of the proposed threshold-adaptive LIF neuron.

The proposed threshold adaptive LIF neuron is shown in Figure 9. Since the threshold voltage of each layer in the DiSNN is specifically set, we employ a Single-Pole-Four-Throw Switch to produce different threshold voltages. Suppose that the voltage *V*_5_ is high in the initial condition. The input current *I_in_* coming from the WCMA circuit will charge the capacitor *C_m_* to simulate the process of charge integration on the biological neuron membrane. The transistor M_1_ plays the role of leakage resistance, which discharges *C_m_* slowly and its leakage rate is decided by *V_l_*. As the input current continues to flow in, the voltage across the capacitor continues to rise non-linearly. Once *V_m_* surpasses the threshold voltage that is decided by transistors M3, M4, M5, and M6, both M5 and M6 will be turned on and pull down the voltage *V_5_*. The inverter composed of M10 and M11 then converts V5 to a high-level voltage at the output port. To reset the LIF circuit, V5 is fed back to the input port through the transistor M2, so that the integration capacitor *C_m_* is discharged quickly, and the discharge rate is controlled by *V_c_* (Yang et al., 2020). A spike will be fired before the neuron is reset. Finally, the LIF neuron enters the refractory period.

## Results and Discussion

### Evaluation of Network Performance

To evaluate the performance of the proposed DiSNN, the MIT-BIH dataset is used in this work (Atzori et al., 2014). The MIT-BIH dataset is separated into the training set and the testing set randomly with the ratio of 4:1. In order to train or infer with the proposed DiSNN, the input data need to be converted into the time dependent spike sequences. The input size of ECG samples during training is 1 × 251 with a batch size of 25, which is min-max normalized into the range of [0,1]. The corresponding spike sequences are generated using the spike encoder.

In this experiment, when the time step of spike-encoder is set to 25, the accuracy of the proposed DiSNN reaches 80, 90, and 95% at the 9th, the 32th, and the 75th epoch, respectively. After training 100 epoches, the proposed DiSNN achieves an accuracy of 93.6% while the computation complexity is reduced by over 92% with only a decline of 4% in accuracy compared to the CNN topology of the same structure, as calculated in Eqs 18, 19.


(18) $$T_{CNN}=\sum M_{H}M_{W}\left(K_{H}K_{W}+K_{H}+K_{W}-1\right)C_{in}C_{out}\times Ops\times bit+\sum N_{in}N_{out}\times Ops\times bits$$

(19) $$T_{SCNN}=\sum M_{H}M_{W}\left(K_{H}+K_{W}-1\right)C_{in}C_{out}\times Ops\times bit\times t+\sum N_{in}N_{out}\times Ops\times bits\times t$$

where *T*, *M*, *N*, *K*, *C*, *H*, *W*, stand for *Time Complexity*, *Feature Map Size*, *Neuron Count in FC Layers*, *Kernel*, *Channel*, *Height*, and *Width*, respectively; *Ops* stands for the needed cycles in one computing operation (1 for adding, 1 for conditional branching, and 10 for multiplication); *bit* stands for the bit number of data flowing between two layers in NN; *t* stands for the number of time steps.

### Effect of Array Size and Memristance Fluctuation on Network Performance

**FIGURE 10:**
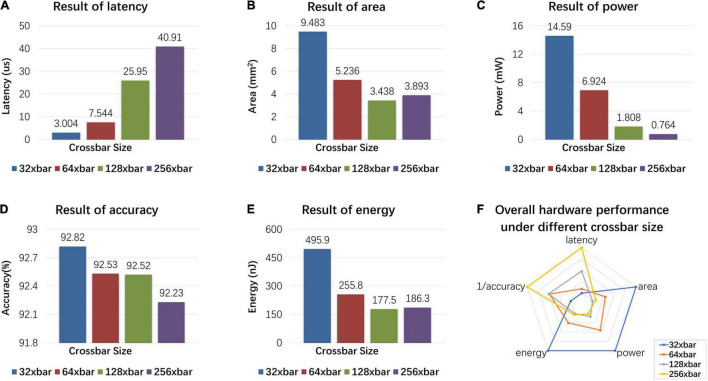
Hardware simulation results: influence of different crossbar sizes on latency, area, power, energy, and accuracy of the proposed MSPAN system. **(A)** The latency of MSPAN system with different crossbar sizes; **(B)** the area of MSPAN system with different crossbar sizes; **(C)** the power of MSPAN system with different crossbar sizes; **(D)** the accuracy of MSPAN system with different crossbar sizes; **(E)** the energy of MSPAN system with different crossbar sizes; **(F)** radar plot of the overall hardware performance under different crossbar sizes.

The proposed MSPAN system is evaluated on the NeuroSim platform (Peng et al., 2020). The results are evaluated at the 65 nm CMOS technology node and the IO bus is designed to work at a clock frequency of 500 MHz. To find out the suitable crossbar size for optimal performance, we examine the influences of crossbar size on latency, area, power, energy, and accuracy of the proposed MSPAN system, as shown in Figure 10.

As the result shows, the latency of hardware system rises as the size of the sub-array increases, which can be ascribed to the reduction of parallelism degree. The area, power, and energy also tend to decrease as the size of the sub-array increases, because the weight matrix needs to be divided into smaller sub-arrays, which results in larger overhead for additional peripheral circuits (such as WCMA circuits and LIF neurons). However, for large crossbar size, non-ideal factors such as IR drop as well as the manufacturing defects are more likely to be introduced, therefore a trade-off is needed between the hardware performance and the computation accuracy for synaptic array.

To evaluate the overall impact of array size on the hardware indicators, a radar chart is drawn intuitively in Figure 10F. The five indicators are linearly mapped to the radar chart, and the largest value of each indicator is mapped to the fifth circle. For the pentagons formed by different array sizes, the smaller the area, the better the overall performance. Therefore, under comprehensive consideration, 128 × 128 is selected as the crossbar size of synaptic array.

**FIGURE 11:**
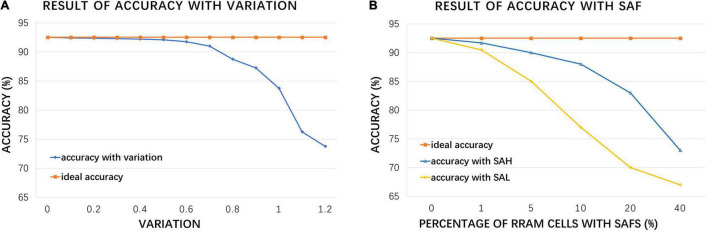
Hardware simulation results: **(A)** accuracy under increasing RRAM device variation compared with ideal accuracy; **(B)** accuracy with increasing RRAM stuck at fault (SAF) rate, where SAH represents stuck at HRS and SAL represents stuck at LRS.

The above simulation result is based on the premise that RRAM is an ideal device, but in fact, due to the limitations of fabrication technology and unstable usage environment, RRAM devices have various non-ideal factors (Chen et al., 2017). To make the simulation result more approximate to the measurement data after taping out, the inference accuracy is re-simulated considering the conductance variation and stuck-at-faults (SAFs) problems, as shown in Figure 11.

For the conductance variation, we focus on device-to-device (d2d) variation and cycle-to-cycle (c2c) variation, which may have great impacts on the accuracy of CIM computation and the performance of DiSNN. Figure 11A demonstrates that the accuracy of the proposed hardware structure will gradually decrease as the variation (estimated as the ratio of conductance fluctuation range to the average conductance) increases, and the decline rate will increase sharply if the ratio is greater than 0.5. Typical fabrication process and room-temperature test environment for inference can restrict the variation parameter lower than 1.0 (Zhang et al., 2019), and the accuracy will drop by 8.49%.

Stuck-at-fault is another common problem that affects the performance of RRAM crossbar chip. It is mainly caused by the defects introduced in the manufacture process, which will result in a certain percentage of RRAM cells to remain in HRS or LRS, i.e., SAF. The device in fault may be randomly distributed in the crossbar structure or assembled in the form of a whole row or a whole column. Figure 11B shows the accuracy with increasing percentage of RRAM cells of SAF. Previous work (Chen et al., 2017) claimed that, the stuck at LRS and stuck at HRS problems are supposed to affect 1.75 and 9.04% of RRAM cells in crossbar array, respectively. Based on such SAF level, the hardware accuracy rate is only reduced by 4.25%, and the entire system still keeps a high performance.

### Simulation With Different Proportions of Neuron Configuration

The LIF circuit in Figure 9 is simulated by Cadence Virtuoso. All transistors in the circuit except the two inverters are supposed to operate in the sub-threshold region. In the simulation process, Cm is set to be 0.9 pF, the W/L ratio of PMOS is 220/100 nm, the W/L ratio of NMOS is 120/100 nm, and *VDD*, *V*_*c*_, *V*_*I*_, *V*1, *V*2, *V*3, and *V4* are set to be 1 V, 850 mV, 300 mV, 900 mV, 950 mV, 1 V, and 1.05 V, respectively. The threshold voltage of the proposed LIF neuron can be adjusted to accommodate the hyperparameter in the DiSNN algorithm.

**FIGURE 12:**
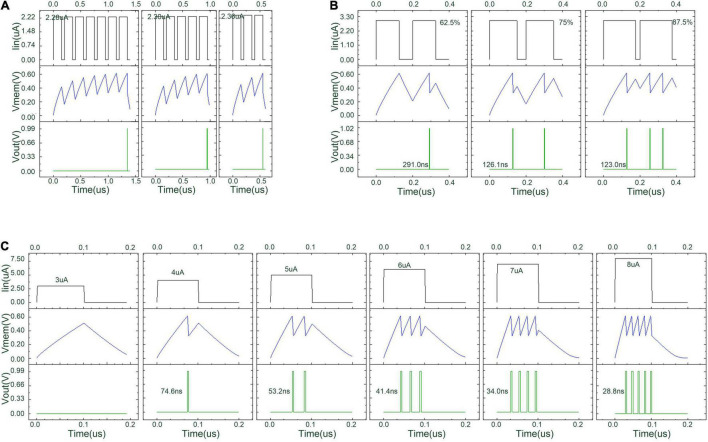
Simulation result of the proposed threshold-adaptive LIF neuron under different input condition. **(A)** The emission time of a LIF neuron under a series of square waves with slightly different amplitudes; **(B)** the output of a LIF neuron under a series of square waves with the same amplitude but increasing duty cycles; and **(C)** the emitted pulse number of a LIF neuron under a series of square waves with increasing amplitude at equal intervals.

Figure 12 shows the simulation waveform of the proposed threshold-adaptive LIF neuron. The three groups of graphs in Figure 12 correspond to different input conditions. In each group, the top line is the input waveform, the middle line is the potential difference across capacitor Cm, i.e., the neuron’s membrane accumulative voltage, and the bottom line is the waveform of the output pulse changing with time. The simulation results indicate that with the increase of input current amplitude, frequency, and duration, the time interval between the emitted pulses of the proposed LIF neuron becomes smaller while the frequency of the emitted pulses also increases.

### Implementation Overheads

**FIGURE 13:**
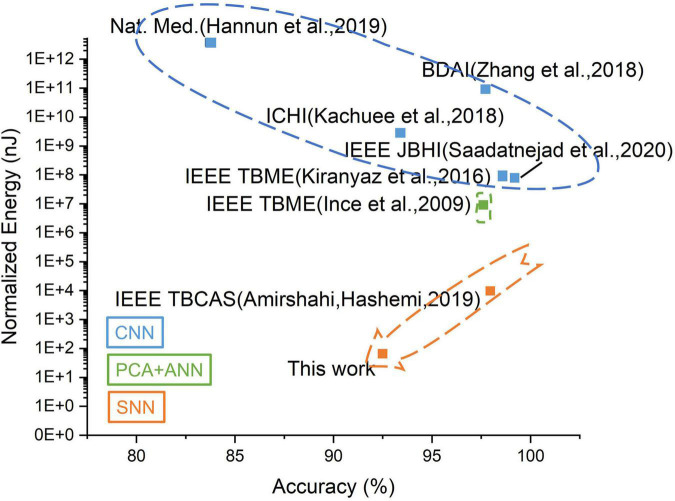
The tradeoff between accuracy and energy for related ECG detection works. The energy has been normalized to 65 nm for a fair comparison.

For AI based edge systems, a tradeoff exists between the energy and the computation accuracy. As shown in Figure 13, the SNN-based systems show overwhelming advantages in energy consumption compared to CNN/ANN-based work while maintaining relatively high accuracy. Further optimization can be achieved by deploying SNN in CIM architecture. The proposed MSPAN system deploying a 7-layer network achieves the lowest energy consumption compared with all counterparts using 65 nm technology.

To demonstrate the contributions of this work, the evaluation metrics including area, energy consumption, and detection accuracy are estimated and compared with other state-of-the-art work, as shown in Table 2. The proposed system exhibits at least 10× energy efficiency compared with other works and also achieves a satisfying accuracy of over 93% in the 4-class ECG classification tasks.

**TABLE 2:** Overall comparison with other state-of-the-art works.

References	Technology (nm)	Area (mm^2^)	Energy (/heartbeat)	Task type	Detection accuracy (%)*	Network type	Computing architecture	Device type
TBME (Kiranyaz et al., 2016)	28	Arm cortex	37 mJ	2-class	98.6	CNN	Out memory	CMOS
ICHI (Kachuee et al., 2018)	28	Arm cortex	1.17 J	4-class	93.4	CNN	Out memory	CMOS
JHBI (Saadatnejad et al., 2020)	28	Arm cortex	35 mJ	4-class	99.2	LSTM	Out memory	CMOS
TBCAS (Amirshahi and Hashemi, 2019)	28	–	1.78 μJ	4-class	97.9	SNN	Out memory	CMOS
IET (Wu et al., 2020)	55	4	1.99 μJ	3-class	97.8	SRNN	Out memory	CMOS
This work	65	3.44	0.178 μJ	4-class	93.6	DiSNN	In memory	RRAM

*The detection accuracy for each work on this table is evaluated at the software level.

## Conclusion

In this work, a memristive spike-based computing engine with adaptive neuron for edge biomedical application is proposed and evaluated on ECG-based arrhythmia detection tasks. A hardware-friendly DiSNN, named DiSNN is first put forward, which can achieve an accuracy of 93.6% on MIT-BIH dataset while the computation complexity is reduced by over 92% with a merely 4% decline in accuracy compared to the CNN topology of the same structure. To deploy DiSNN in edge devices for inference, a memristor based CIM architecture is implemented. The proposed DiSNN achieves a satisfying detection accuracy of 92.25% and the average energy consumption is only 0.178 μJ per heartbeat at a supply voltage of 1.0 V and a working frequency of 500 MHz in 65 nm technology. The low energy consumption greatly surpasses related works in ECG-based arrhythmia detection field.

## Data Availability Statement

Publicly available datasets were analyzed in this study. This data can be found here: https://physionet.org/content/mitdb/1.0.0/.

## Author Contributions

JJ and FT designed the architecture of memristive spike-based computing engine and all the relevant experiments. FT preprocessed the dataset and designed the model structure. FT and JL designed the DiSNN and conducted the software experiments. JJ designed the CIM architecture and the computing pattern. JJ and YL designed the LIF neuron circuit. JJ, YL, and HW conducted experiments on the neuron circuit. JJ, FT, JZ, ZS, YL, HW, ZZ, CF, YZ, and JS contributed to experiment data collection and the writing of the manuscript. XX supervised this study. All authors discussed the results.

## Conflict of Interest

The authors declare that the research was conducted in the absence of any commercial or financial relationships that could be construed as a potential conflict of interest.

## Publisher’s Note

All claims expressed in this article are solely those of the authors and do not necessarily represent those of their affiliated organizations, or those of the publisher, the editors and the reviewers. Any product that may be evaluated in this article, or claim that may be made by its manufacturer, is not guaranteed or endorsed by the publisher.
